# THE VIRGINIA MEMORY PROJECT: AN INNOVATIVE SERVICE AND EPIDEMIOLOGICAL BRAIN HEALTH REGISTRY

**DOI:** 10.1093/geroni/igad104.2760

**Published:** 2023-12-21

**Authors:** Ann Rhodes, Rachel Coney, Melicent Miller

**Affiliations:** Virginia Commonwealth University, Richmond, Virginia, United States; Virginia Department of Health, Richmond, Virginia, United States; Division of Prevention and Health Promotion: Chronic Disease Unit, Richmond, Virginia, United States

## Abstract

The Virginia Memory Project (VMP) is a statewide memory loss and caregiving needs registry in Virginia, United States. The VMP was launched in June of 2022 and supports statewide surveillance efforts via expanding the BRFSS (Behavioral Risk Factor Surveillance System) memory loss and caregiving modules via a digitized, mobile-capable online survey. In addition to collecting BRFSS data, the VMP partners with regional community brain health organizations to support respondents in getting connected to brain health and caregiving services. The VMP model is innovative because it combines a service and epidemiological registry to support improved resource allocation and decision-making in Statewide dementia care planning. It also provides a pathway for caregiving and memory loss services. In the pilot period of the VMP, there were 138 respondents, with a median age of 61, mean=58, range=18-100, and most respondents identified as White 65% (n=99), with smaller proportions of Black/AA, 16.7% (n=22), and Asian 5.3% (n=7). 96.2% of respondents were non-Hispanic. Most respondents were married (n=66, 50.4%) with no children in the home (77.9%), and 26.7% (n=35) provided contact information for potential brain health or caregiving services. For respondents who completed the memory loss module, 33% (n=26) reported memory loss or confusion worsening over the last year, but only 60% (n=15) had discussed symptoms with a healthcare professional. 33.3% (n=24) reported having caregiving responsibilities. The VMP increases brain health surveillance capacity in Virginia and represents an innovative pathway between a state department of health, a university data registry, and a community service provider.

